# London and Counties Medical Protection Society

**Published:** 1915-05-22

**Authors:** 


### LONDON AND COUNTIES MEDICAL PROTECTION SOCIETY.

### The Profession and its Safeguards.

The issue of the Report of this Society affords an opportunity for drawing the attention of members of the medical profession to the important work which such organisations undertake, and to the advisability of securing the benefits they are able to offer. In consequence of the nature of medical work—its privacy, responsibility, and liability to misconstruction—practitioners are particularly exposed to the risk of misrepresentation, sometimes the consequence of ignorance, sometimes due to deliberate malice. There is, therefore, corresponding need on their part for self-protection, and manifestly this can be more effectually secured by mutual co-operation than by individual effort. An organisation devoted to this end has several advantages. It accumulates a body of experience and tradition which can be applied through its officials for the benefit of any member in his individual difficulties. Upon all occasions it is a refuge where skilled advice and direction may be secured. If necessity arises to speak with the enemy in the gate, the voice of an organisation is likely to be both more wise and more effective than the speech of any isolated unit. Last, but by no means least, membership secures the peace of mind which comes from the knowledge that the remedy of the Law Courts, should this be necessary, can be contemplated without personal financial anxiety. In short, a medical protection society offers for a small annual subscription the counsel, guidance, and assistance which are necessary to the practitioner in times of trouble.

In view of the advantages just stated, it is surprising that any practitioner should remain outside the shelter which such organisations offer. Only too often, however, procrastination governs conduct, and a loose conviction that all will continue to be well delays action. Then suddenly, like a bolt from the blue, comes trouble, and there is at hand no efficient helper. Such experiences occur almost daily, and yet the warning is not heeded. All men, it is said, think all men mortal but themselves, and a similar principle leads to negligences in the direction here considered. Hence we think it a duty now and again to draw the attention of our readers to the risks they run so long as they remain outside these organisations. To say this is an old story, yet the comparatively small membership of these organisations shows the necessity of repeating it. More than once we have urged on the teachers in our medical schools their responsibility in this matter. Every student ought to be urged most strongly to secure protection immediately he receives his qualifying diploma; and, considering the nature of the subject he teaches, the responsibility for giving this advice would seem to rest more particularly with the lecturer on forensic medicine. Part of his duty is the discussion of the public relations and legal duties of the practitioner, and he might well undertake the task of indicating the risks which these involve and the manner in which these risks may most prudently be met. To leave a student ignorant of the importance of these matters may reasonably be condemned as a neglect of duty.

To turn to the Report now before us: Let us take a common experience in the shape of charges of unskilfulness or of negligence. Some of these are due to disappointment or to ignorance; others are advanced in order to avoid the payment of fees. In either case they are liable to be very hurtful to the practitioner concerned, and whatever explanation he may give is very apt to be regarded as the excuse of an interested or incriminated person. Only too frequently, it is to be feared, the practitioner, rather than take up a contentious attitude, will renounce his just rights, with the inevitable result that colour is given to the slander. Very different is the situation when the matter is handled by the experienced officials of a professional organisation. It is possible for these to gain a hearing where an immediately interested voice is of no avail. Honest blunders and misunderstandings can be corrected, and a true conception of the position can be secured. Again and again it happens that patients, or the relatives of patients, angry, hurt, and wrong-headed, are led to see their errors, and are willing to express regret for the misrepresentations of which they have been guilty. In another direction, the wilful slanderer, who hopes by the malignancy of his tongue to avoid payment of money due, is readily brought to silence—and payment—when he realises that he is in the grip of an organisation which is well accustomed to deal with creatures of his kidney. Let any man compare the contrasts thus presented if he wishes to realise the advantages so easily secured.

There is one point about the manner of utilising the help of such societies—namely, the importance of taking advice and direction at the very beginning of any dispute. There are some matters which a sensible man will ignore, as, for example, mere idle gossip and tittle-tattle. But if he feels that serious and deliberate statements prejudicial to his personal character or his professional competence are being made, let him not hesitate to take decided action. In doing so he will be wise to act under advice from the very first step. He may, indeed, listen to counsel as to whether he should act at all, but in no circumstances ought he to commence a personal controversial correspondence. With the best intentions in the world he may entangle the issue and perhaps say or do something which proves to be much to the prejudice of his own position. Given his membership he has efficient aid at hand; he will be wise to take full advantage of this rather than to risk a later presentation to the society of a case which, by his personal action, has become complicated and involved. In a word, we would urge every member of the profession to become a member of a protection society and then to utilise fully the guidance thus rendered accessible.

